# Triclosan in water, implications for human and environmental health

**DOI:** 10.1186/s40064-016-3287-x

**Published:** 2016-09-21

**Authors:** L. W. B. Olaniyan, N. Mkwetshana, A. I. Okoh

**Affiliations:** 1 SAMRC Microbial Water Quality Monitoring Centre, University of Fort Hare, Private Bag X1314, Alice, Eastern Cape 5700 South Africa; 2 Applied and Environmental Microbiology Research Group (AEMREG), Department of Biochemistry and Microbiology, University of Fort Hare, Alice, 5700 South Africa

**Keywords:** Cytotoxicity, Endocrine disruptor, Micro-pollutant, Triclosan, Water

## Abstract

Triclosan (TCS) is a broad spectrum antibacterial agent present as an active ingredient in some personal care products such as soaps, toothpastes and sterilizers. It is an endocrine disrupting compound and its increasing presence in water resources as well as in biosolid-amended soils used in farming, its potential for bioaccumulation in fatty tissues and toxicity in aquatic organisms are a cause for concern to human and environmental health. TCS has also been detected in blood, breast milk, urine and nails of humans. The significance of this is not precisely understood. Data on its bioaccumulation in humans are also lacking. Cell based studies however showed that TCS is a pro-oxidant and may be cytotoxic via a number of mechanisms. Uncoupling of oxidative phosphorylation appears to be prevailing as a toxicity mechanism though the compound’s role in apoptosis has been cited. TCS is not known to be carcinogenic per se in vitro but has been reported to promote tumourigenesis in the presence of a carcinogen, in mice. Recent laboratory reports appear to support the view that TCS oestrogenicity as well as its anti-oestrogenicity play significant role in cancer progression. Results from epidemiological studies on the effect of TCS on human health have implicated the compound as responsible for certain allergies and reproductive defects. Its presence in chlorinated water also raises toxicity concern for humans as carcinogenic metabolites such as chlorophenols may be generated in the presence of the residual chlorine. In this paper, we carried out a detailed overview of TCS pollution and the implications for human and environmental health.

## Background

Triclosan (TCS) is a broad-spectrum antimicrobial agent in some personal care products such as soap, sanitizer and skin cream (Kirk–Othmer 1993; MacIsaac et al. 2014; Perencevich et al. 2001; Schweizer 2001). Its widespread use in homes and in health care centres may have explained its versatility as a water micro-pollutant (Helbing et al. 2011; Kolpin et al. 2002; Li et al. 2010; Loraine and Pettigrove 2006; Park and Yeo 2012; Reiss et al. 2002). TCS has been mentioned (Foran et al. 2000) as an endocrine disruptor (ED), a group of compounds known to interfere with hormone functions (Wingspread Consensus Statement 1991).

**Fig. 1:**
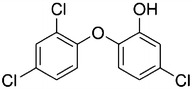
Structure of triclosan (CAS 3380-34-5)

Structurally, TCS molecule possesses functional groups for both phenol (5-chloro-2-(2,4-dichlorophenoxy) phenol) and ether (2,4,4-trichloro-2-hydroxydiphenyl ether) (Fig. 1). Its lipophilicity (log K_ow_ = 4.8 octanol–water partition coefficient) suggests bioaccumulation in fatty tissues which raises toxicity concern.

The hormonal activity of TCS (Crofton et al. 2007) is widely acknowledged in vitro (Huang et al. 2014) and in vivo, in laboratory (Stoker et al. 2010) and aquatic (Ishibashi et al. 2004) animals which raises fear for human health. Evidence of human toxicity is still a matter for debate in scientific circles, however some state authorities such as in Minnesota are considering a ban on all TCS-containing products (Dhillon et al. 2015) or a restriction (TIME 2014), partly due to the reported TCS-resistant bacteria or ostensibly yielding to the pressure mounted by some professional bodies (APUA 2011b) for the perceived adverse effects on health. Apart from its widely reported adverse effects on cellular metabolism, its ecotoxicity also raises fear of dwindling economic fortunes from water resources. The present work attempts to review available data on environmental impact as well as evidence suggestive of human toxicity and to suggest future research directions.

## Triclosan exposure for humans

TCS is approved for topical administration at maximum concentration of 0.3 % (w/w) in humans (European Commission 2010; Larsson et al. 2014; MacIsaac et al. 2014) and 0.03 % (w/w) in oral medications in some countries (Canada 2007). It is used as an additive in polymer matrices such as polyolefin and polyethylene which may contain 1 % (w/w) to 10 % (w/w) TCS in some plastics industries (NICNAS 2009). Current annual global TCS production is not known to us but as many as are using the over 200 TCS-containing products as well as workers where these products are manufactured risk TCS exposure. Previous reports estimated over ‘1500 tons’ of TCS per year entering consumer markets globally (Singer et al. 2002) and estimated value of well over 1.1 × 10^5^ kg/year of TCS have been released into wastewater in the United States (Heidler and Halden 2007). Direct application of TCS-based products such as soaps, deodorants and toothpastes is the primary source of human exposure to TCS (Allmyr et al. 2006; Fang et al. 2014; MacIsaac et al. 2014). Occupational and environmental exposures have also been documented in humans (Geens et al. 2009). Among workers, dermal as well as inhalation are the routes of exposure. TCS bioavailability via inhalation is thought to be 100 % (NICNAS 2009) but toxicokinetic data from this exposure route are lacking.

Ability of certain seafood to accumulate TCS is a route of TCS exposure to humans (Adolfsson-Erici et al. 2002). Rüdel et al. (2013) recorded maximum TCS of 11.7 nmol/kg in fish. Certain plants can also accumulate TCS in their tissues (Pannu et al. 2012) including plant foods such as lettuce (Prosser and Sibley 2015) which may occur following their cultivation in soils amended with biosolids, manure or irrigated with wastewater. For example Pannu et al. (2012) reported triclosan in radish at 31.8 µmol/kg dry weight (dw) when the plants were grown in soil with TCS concentration of 34.2 µmol/kg dw. However such accumulation becomes something of toxicological concern to humans (hazard quotients ≥ 0.1) (Health Canada and Environment Canada 2012; US FDA 2010) if the accumulation has been stored in the edible portions of the plant (Aryal and Reinhold 2011; Wu et al. 2012a) though it has been recorded that TCS translocation from the plant roots can reach the above-ground parts (Wu et al. 2010).

In infants exclusively on breastfeeding, breast milk presents major route of exposure to TCS (Table 1). In highly industrialized countries such as USA, TCS concentration in breast milk as high as 2.1 mol/kg lipid has been reported (Dayan 2007) which should be of health concern to the young because of their immature drug metabolizing pathways making them vulnerable to the impact of TCS. On average, TCS concentration in drinking water is below parts per billion (Table 2) showing that it may not be a quantitative source of TCS exposure to humans.

**Table 1:** Infant exposure to triclosan via breast milk (NICNAS 2009)

Age (month)	Average milk intake (g/day)	Body weight (kg)	Internal triclosan dose (nmol/kg body wt/day)
1	751	4.7	10
2	725	5.6	8.5
3	723	6.2	7.6
4	740	6.7	7.3

**Table 2:** Triclosan concentrations in drinking water

Source	Concentration (nM)	Frequency-of detection (%)	References
Tap water	0.17	34	Yang et al. (2014)
	0.048	75	
Tap water	0.21	63	Padhye et al. (2014)
Tap water	0.021–0.052		Li et al. (2010), Perez et al. (2013)
Fountain water	0.36	45	Yang et al. (2014)
	0.028	75	

The estimated acceptable daily intake for TCS is 0.17 nmol/kg/day (Blanset et al. 2007) and some drinking water levels of TCS have been found to be higher than this estimate (Table 2).

TCS after disposal, drains ultimately into underground (Sorensen et al. 2015) and to surface waters (Table 3) where it may finally reach humans by drinking contaminated water or via the food chain such as consumption of animals and vegetation exposed to TCS (Park and Yeo 2012). An annual discharge of 18 tonnes of TCS to the surface water in USA had been reported, with more than half this volume coming from wastewater treatment plants (WWTPs) (Halden and Paul 2005). Underground water from shallow wells and boreholes has been reported to contain up to 0.10 nM TCS (Sorensen et al. 2015). Concentrations of TCS in some untreated surface waters were reported to range from 7.9 to 39 nM (Kolpin et al. 2002; Perez et al. 2013). High value of 297.7 nM was reported in the influents of certain wastewater treatment plants (Kumar et al. 2010) while effluents concentrations ranging from 0.41 to 3.5 nM were reported in treated wastewater effluents (Glassmeyer et al. 2005; Snyder et al. 2008).

**Table 3:** Triclosan concentrations in aquatic systems

Source	Concentration (nM)	References
*Surface water*		
Natural streams/rivers	nd–7.9	Ying et al. (2007)
	0.26	Halden and Paul (2005)
Streams with input of raw wastewater	5.5	Fair et al. (2009)
Ebro basin (Spain)	nd–0.98	Kantiani et al. (2008)
Danshuei River (Taiwan)	0.015–0.036	Shen et al. (2012)
River (SouthWest Spain)	0.25 ± 0.0017	Pintado-Herrera et al. (2014)
Sea (SouthWest Spain)	0.23 ± 0.0034	Pintado-Herrera et al. (2014)
*Undergroundwater (boreholes/wells)*		
South West Spain	0.23 ± 0.0035	Pintado-Herrera et al. (2014)
Kabwe, Zambia	6.9 × 10^−5^–1.0 × 10^−4^	Sorensen et al. (2015)
West Texas USA	nd–0.18	Karnjanapiboonwong et al. (2011)
*Wastewater-treatment plants*		
Treated wastewater (Midlands, UK)	Mean 0.25–1.51	Chi et al. (2013)
Influent (USA)	6.91–10.36	Anumol and Snyder (2015)
Final effluents (USA)	0.044–0.097	Anumol and Snyder (2015)
Effluent (South West Spain)	0.33 ± 0.028	Pintado-Herrera et al. (2014)

*nd* not detected

## Triclosan absorption in humans

TCS absorption in humans and animals can be through dermal (Fang et al. 2014; Moss et al. 2000; Queckenberg et al. 2010), mucous membranes of the oral cavity (Lin 2000) or gastrointestinal tract (Bagley and Lin 2000) routes and reaches systemic circulation (Hovander et al. 2002). It penetrates the skin less rapidly and less extensively in humans than in rats (Chedgzoy et al. 2002; Moss et al. 2000), a factor to consider in risk assessment. Animal experiments had shown that tissue levels of TCS are less when administered through dermal route than via oral route. Percutaneous absorption is interfered with by the vehicle of administration. Propylene glycol increases percutaneous absorption of TCS in mice however no such change is observed when 95 % ethanol is used (Fang et al. 2014).

TCS absorption and distribution are rapid in humans. Dermal absorption is generally 3–7 % (Moss et al. 2000; NICNAS 2009; Queckenberg et al. 2010), 6.3 % was reported in vitro using human skin (Moss et al. 2000). Lin (2000) reported that 7.33 % TCS was retained from mouthwash containing 0.03 % TCS but when swallowed the absorption was higher at the gastrointestinal tract (Bagley and Lin 2000) which has been reported to be near-completion (NICNAS 2009). Maximum blood level was reported to have been reached when exposed orally within 3 h while significant amount of the dose was excreted 24 h post-dose (Sandborgh-Englund et al. 2006). Studies carried out on human subjects using TCS-containing cosmetics showed variable but significant amounts of TCS in their body fluids (Table 4) when compared with the unexposed controls (Allmyr et al. 2006; Sandborgh-Englund et al. 2006). A single oral dose of 13 µmol TCS to human subjects increased plasma TCS level to 0.84 µM 4 h after ingestion and when repeated thrice daily for 12 days increased the plasma level to 1.2 µM (Bagley and Lin 2000) but administration of 14 µmol TCS per oral increased the plasma level to 0.75 µM, 3 h post-dose (Sandborgh-Englund et al. 2006). The results are not comparable because of certain militating factors which included sample size and inter-subject variation. Before entry into the blood stream, TCS is conjugated by the skin (Moss et al. 2000). Both percutaneous and gastrointestinal tract absorptions are susceptible to first-pass metabolism wherein TCS is conjugated with glucuronates and sulphates (Moss et al. 2000), the process known to be the cellular means of detoxication. Toxicokinetic data on TCS oral and dermal administrations in humans appear limited. In a controlled exposure study, some 70 % of the total plasma TCS are in conjugated form (Sandborgh-Englund et al. 2006) and would be voided predominantly via urine, the balance is in unconjugated form expected to evoke physiological responses. However, from data presented by Allmyr et al. (2009), the 30 % balance equal to 0.03–0.08 µM TCS in the plasma failed to activate pregnane X receptor (hPXR) regardless of the high level of exposure.

**Table 4:** Triclosan concentrations in human fluids

Fluid	Concentration (nM)	Location	References
Serum	4.1–41.4	Spain	Azzouz et al. (2016)
Plasma	0.035–1200	Australia, Sweden	Allmyr et al. (2006, 2008)
Urine	8.3–13,090	USA	Calafat et al. (2008), Allmyr et al. (2008)
	0.56 ± 1.8^a^	India	Xue et al. (2015)
	0.16 ± 0.27^b^	India	Xue et al. (2015)
	1630	USA	US CDC (2014)
	1.1–7.3	Spain	Azzouz et al. (2016)
	0.51 ± 0.53	USA	MacIsaac et al. (2014)
Breast milk	0.86–7.3	Spain	Azzouz et al.( 2016)
	0.062–252	USA, Australia, Sweden	Allmyr et al. (2008), Adolfsson-Erici et al. (2002)

^a^Non-obese

^b^Obese

In addition to blood, detectable levels of TCS have been recorded in breast milk (Dayan 2007) and urine (Benny et al. 2014; Table 4). Azzouz and colleagues (2016) reported 41.4, 7.3 and 7.3 nM in whole blood, breast milk and urine respectively as maximum TCS concentrations from healthy humans. In highly industrialized nations such as U S, about two-thirds of 90 girls, aged 6–8 years old have been reported to exhibit detectable urinary TCS ranging from 5.5 nM to 3.3 µM (Wolff et al. 2007). Among some Chinese school children aged 3–24 years old, higher TCS levels in urine (geometric mean of 13 nM) were reported among females than their male counterparts, significantly (93 % detection frequency) among those between 18 and 24 years (Li et al. 2013). It is presently not clear if gender plays a definitive role in TCS metabolism as reports by Clayton et al. (2011) from National Health and Nutrition Examination Survey (NHANES) among US population aged 50 years had shown that males had higher urinary TCS than females (472.34 vs. 329.42 nM) whereas data of Yin et al. (2015) from respondents (<50 years) containing equal number of both sexes reported higher geometric mean for urinary TCS among females than males (1.83 nmol/g creatinine vs. 1.1 nmol/g creatinine). The data from US CDC (2014) however appeared not in support of this observation.

## Metabolism of triclosan

TCS is readily metabolized and may be extensively distributed in tissues including the brain (Geens et al. 2012) showing its ability to cross blood–brain barrier. It induces cytochrome P450 in rat hepatic microsomes, the enzymes responsible for its metabolism (Hanioka et al. 1997; Kanetoshi et al. 1992). It undergoes glucuronidation and sulphation in animals and humans (Sandborgh-Englund et al. 2006; Wang et al. 2004) regardless of the route of administration. In mice, TCS is metabolizable into sulphate, glucuronide, 2,4-dichlorophenol, and hydroxyl derivatives. Small amount (0.36 %) of unconjugated TCS has been detected in the blood plasma when administered intraperitoneally (Cherednichenko et al. 2012). In rats as well as in humans, topical application of TCS on the skin generates a fraction of the sulphate conjugation product of the compound leaving higher amount unconjugated (Moss et al. 2000). In rats and mice, free TCS is excreted in the bile and voided via faeces (Fang et al. 2014).

TCS may inhibit the activities of the conjugating enzymes, glucuronyltransferases and sulphotransferases, towards 3-hydroxybenzo(α)pyrene, acetaminophen and bisphenol A in human liver microsomes (Wang et al. 2004) possibly blocking the elimination of these compounds and at the same time increasing their toxicities when co-administered. Glucuronate conjugation predominates in humans and is secreted in urine along with the unconjugated specie (Arbuckle et al. 2015; Benny et al. 2014; Weiss et al. 2015). Elimination half-life for TCS is higher in humans than in the laboratory animals such as rats (for example, 13–29 h in humans, 25–32 h in hamsters, 10–15 h in rats and 8–12 h in mice) when orally administered (NICNAS 2009). Elimination half-life is related to drug toxicity. Based on this, it is expected that TCS should be more toxic when applied under similar conditions to rats than humans and this needs be taken into consideration in human risk assessment using animal data.

## Endocrine disruption activity and toxicity of triclosan

Mechanism of endocrine disruption by exogenous agents can take many forms (Colborn et al. 1993; Golden et al. 1999; Hollander 1997; Solomon and Schettler 2000; Wingspread 1991), the commonly encountered is the inhibition of the hormone (agonist) from binding its receptors by competing for the receptor binding sites with the antagonist. This is one mechanism through which TCS exhibits its endocrine disruption activity (Ahn et al. 2008; Gee et al. 2008; Jung et al. 2012). The occupation of the receptor site by a ligand is known to induce conformational change in the receptor leading to the generation of the transcription factors required for the expression of the hormone-sensitive genes. The expression of oestrogen sensitive genes through the antagonist leads to various uncontrolled physiological effects, such as hypospadia, cryochidism and cancer (Meng 2005). TCS oestrogenic, anti-oestrogenic, androgenic and anti-thyroid activities in vitro and in vivo in laboratory and aquatic animals have been demonstrated (Arancibia et al. 2009; Crofton et al. 2007; Henry and Fair 2013; Jung et al. 2012; Schiffer et al. 2014; Wallet et al. 2013) at environmentally relevant concentrations. Its anti-estrogenic effect in sheep (James et al. 2010) and anti-androgenicity in albino rats (Kumara et al. 2009) have also been demonstrated.

The observed physiological effects such as precocious puberty (Stoker et al. 2010) and carcinogenicity (Lee et al. 2012b) could be explained as a consequence of over-stimulation of the receptors presumably by the high TCS concentration (Henry and Fair 2013) or as a consequence of its occupation of the ligand binding domain of the receptor (Ahn et al. 2008; Gee et al. 2008; Jung et al. 2012; Meng 2005). More data are required relating environmentally relevant TCS concentration with the reported physiological effects such as adverse reproductive effects in animals (Kumara et al. 2009).

Data appear to be accumulating supporting aetiologic role for TCS in carcinogenesis (Lee et al. 2012b; Rodricks et al. 2010; Winitthana et al. 2014; Wu et al. 2014; Yueh et al. 2014). Hepatic tumourigenesis in mice exposed to TCS has been reported to be mediated by peroxisome proliferator-activated receptor α (PPARα) signalling pathway (Rodricks et al. 2010; Wu et al. 2014). But the work of Yueh et al. 2014 in which tumour was promoted in mice exposed to 0.1 mol/kg TCS in drinking water for 8 months following diethylnitrosamine, (a pro-carcinogen) pre-treatment did not activate PPARα in cancer promotion. Additional data would be needed to deny or confirm these contrasting reports. PPARα is a ligand-activated transcription factor belonging to the nuclear receptor superfamily (Corton et al. 2014). It plays a key role in the regulation of lipid metabolism. Its activation by peroxisome proliferators is a well-characterized mode of action of hepatocarcinogenesis in rodents (Corton 2010; Corton et al. 2014). TCS hepatocarcinogenesis via PPARα signalling pathway is not expected in humans because the pathway is known to be several times less active in humans than in mice (Health Canada 2012; US EPA 2008).

The report of Lu and Archer (2005) in which mammary tumour was inhibited in methylnitrosourea-treated rats fed with diets containing TCS may appear contrasting to the previous reports of tumour promoting activity of TCS but actually lends credence to the anti-oestrogenic effect of TCS since the presence of oestradiol is a requirement for developing breast cancer (Fernandez and Russo 2010; Gee et al. 2008; Henry and Fair 2013). But report from more recent studies (Lee et al. 2014) showed that TCS induced-cancer progression in MCF-7 human breast cancer cell occurred via oestrogen receptor-mediated signalling pathway implying that TCS participates through multiple mechanisms in breast cancer progression.

TCS perturbs thyroid homeostasis (Kodavanti and Curras-Collazo 2010; Veldhoen et al. 2006). It reduces circulating levels of the hormones (hypothyroxinaemia) in the exposed animals (Crofton et al. 2007; Petersen et al. 2013). The compound interferes with thyroid‐mediated developmental processes of tadpoles into frogs (Fort et al. 2010, 2011). The effects are expected to be shared by all animals including humans whose cellular metabolism involves thyroid signalling pathway. Multiple mechanisms including induction of phases I and II enzymes through activation of pregnane X receptor are thought to be responsible for the anti-thyroid activity (Hanioka et al. 1996; Jacobs et al. 2005; Jinno et al. 1997; Paul et al. 2010). Sodium/iodide symporter is the protein normally responsible for iodide uptake but its role in this scenario has not been defined (Friesema et al. 2005; Paul et al. 2010).

TCS toxicity has been demonstrated in a number of cells including human live and cancer cells (Arancibia et al. 2009; Wallet et al. 2013) exhibiting different toxicities in different cells (Table 5). It is pro-apoptotic at ≥1 nM and cytotoxic at ≥50 µM in human choriocarcinoma-derived placental JEG-3 cell line when exposed for at least 24 h (Honkisz et al. 2012) but may not in certain cells (Weatherly et al. 2016). Its mitochondrial uncoupling activity resulting in the loss of oxidative phosphorylation and consequently reduced ATP generation has been documented in vitro (Ajao et al. 2015, Weatherly et al. 2016) and in vivo (Shim et al. 2016) at micromolar exposure levels. TCS is a pro-oxidant (Ma et al. 2013; Tamura et al. 2012; Yueh et al. 2014). The pro-oxidant activity is thought to be related to the activity of 2,4-dichlorophenol (Gou et al. 2014), one of the TCS chlorinated by-products of photolysis. TCS oxidation of deoxyguanosine has been demonstrated to be inversely related to DNA methylation (Ma et al. 2013), a biomarker for tumour development and progression. The generation of reactive oxygen species is significant in cells such as β cells known for low expression of antioxidant enzymes (Pi et al. 2007) which definitely impacts on insulin synthesis and function and ultimately diabetes pathogenesis.

**Table 5:** Reported triclosan (TCS) toxicities

Cell type	TCS Concentration (µM)	Exposure (h)	Toxicity	References
Human breast cancer cells MCF-7	≥345.4	120	Proliferation (oestrogenicity)	Henry and Fair (2013)
Human (A549) lung cancer cell	250	24	Cell membrane damage (LDH release)	Kwon et al. (2013)
Human (H460) lung cancer cell	10	24	Apoptotic and proliferative effect, Cell membrane damage (LDH release)	Winitthana et al. (2014)
Human PBMC	≥8.6	30	Loss of mitochondrial transmembrane potential; metabolic acidosis; uncoupling of oxidative phosphorylation	Ajao et al. (2015)
Human keratinocytes HaCaT	≥8.6	30	Loss of mitochondrial transmembrane potential; necrosis	Ajao et al. (2015)
Rat fibroblast cells (RBL)	≥5	1	Uncoupling of mitochondrial oxidative phosphorylation	Weatherly et al. (2016)
Human mast cells (HMC-1.2)	≥5	1		
Mouse JB6 Cl 41-5a cells	≥8	≥48	Decreased growth; cell damage (increased LDH release); necrosis	Wu et al. (2015)
Zebrafish (*Danio rerio*, AB strain) embryos	15	1	Uncoupling of mitochondrial oxidative phosphorylation	Shim et al. (2016)
Freshwater Protozoan (*Tetrahymena thermophile*)	3.5 nM	1	DNA damage (20 % DNA)	Gao et al. (2015)
*Chironomus riparius* Larvae.	35 nM	24	DNA damage	Martínez-Paz et al. (2013)

TCS generally demonstrates low acute toxicity in rodents with LD50 values in excess of g/kg body weight (US FDA 2008). There are reports that rats showed pathologic changes in liver and blood when chronically exposed for more than 13 weeks at doses higher than 150 ppm in the diet (US FDA 2008) or in excess of 300 ppm/day by oral gavage after 4 days (Bhargava and Leonard 1996; Crofton et al. 2007; DeSalva et al. 1989). Renal toxicity has also been reported in rats orally dosed 200 ppm/day for 6 weeks (Hassan et al. 2014). The activity of TCS in muscle Ca^2+^ dysregulation in mouse has been investigated (Cherednichenko et al. 2012). Exposure to TCS at 25 ppm (≥0.09 mol/kg) for ≤60 min intraperitoneally was recorded to impair excitation–contraction coupling showing that the compound may be myotoxic.

In vivo human toxicity of TCS has not been precisely demonstrated, but detectable levels of TCS reported in the body fluids such as blood, breast milk and urine of exposed humans (Table 4) as well as in human tissues such as adipose tissue, brain, liver and nails (Table 6) fuel the conception that the compound may possibly impact human physiology. The high TCS concentrations in the tissues (Table 6) relative to the environmental concentrations (Tables 2, 3) may imply that TCS bio-accumulates and extensively distributed in human tissues.

**Table 6:** Tissue distribution of triclosan in humans

Tissue	Concentration (nmol/kg)	References
Liver	10.8	Geens et al. (2012)
Adipose	2.1	Geens et al. (2012)
	80.1 (7.6–80.1)	Wang et al. (2015)
Brain	0.1	Geens et al. (2012)
*Nails*		
Toes	19.6 (nd–3.62 µmol/kg)	Yin et al. (2015)
Fingers	46.9 (nd–17.4 µmol/kg)	

*nd* not detected

There have been reported cases of TCS-induced allergic reactions in human subjects. Dermatitis following prolonged use of TCS-containing hand washes (Wong and Beck 2001) or when further exposed to sunlight after use (Schena et al. 2008) have been recorded. Similarly, blisters were known to have erupted in the mouth and on the lips of human subjects following prolonged use of TCS-containing toothpaste (Robertshaw and Leppard 2007). Epidemiological report has associated the increased TCS levels in urine with immune dysfunction (Clayton et al. 2011), allergic reactions and production of asthma in the children (Bertelsen et al. 2013; Spanier et al. 2014). Laboratory demonstration has shown that TCS is able to interact with human serum albumin resulting in conformational change of the protein (Chen et al. 2012a). The binding of toxicants to serum albumin can impede the transport of endogenous substances and cause conformational changes in the protein molecule, which may affect its activity or even change its physiological function (Qin et al. 2010; Soares et al. 2007). A retrospective study (Vélez et al. 2015) found that elevated TCS in the maternal urine correlated positively with diminished fecundity. Whether or not this result is related to TCS oestrogenicity (Jung et al. 2012) is presently not clear. The adverse effects of TCS in humans are thought to be via the inhibition of fatty acid synthase type 1 (FAS 1) (IC_50_ ≥ 10 µM) and partial inhibition of enoyl reductase of FAS 1 reactions (Liu et al. 2002). The TCS cytotoxic mechanism is being explored for drug target in cancer therapy (Sadowski et al. 2014).

## Fate of triclosan in environmental water

Wastewater treatment plants (WWTPs) are not designed to remove pharmaceuticals or EDs; rather removals are based on the physical and chemical properties of the compounds. The efficiency of WWTPs is measured using parameters, such as biochemical oxygen demand (BOD) and chemical oxygen demand (COD), which do not take into account ED removal. During sewage treatment, EDs are only partially removed and are therefore frequently detected in WWTP effluents (Gomez et al. 2007; Svenson et al. 2003). Consequently, TCS is not completely removed from influents of WWTPs, (Bock et al. 2010; Snyder et al. 2007) or not at all during primary treatment (Lozano et al. 2013) and whatever remains in the aqueous phase is released into the receiving water body which may impact on the aquatic ecosystems.

TCS is stable to hydrolysis; laboratory studies showed it was stable at pH 4, 7 and 9 (US EPA 2008b). TCS is not expected to volatilize significantly given its low vapour pressure of 4 × 10^−6^ mm Hg at 20 °C (Ciba Speciality Chemicals 2003), however it undergoes biodegradation, photolysis and photochemical reactions, which are processes thought to be responsible for its reduction in natural waters. In conventional treatment plants, substantial amount of TCS is removed from wastewater (Table 7) but advanced treatment processes such as ozonation, photolysis and microfiltration/nanofiltration with reverse osmosis (membrane process) have achieved somewhat total removal of pharmaceuticals (Watkinson et al. 2007; Ziylan and Ince 2011) (Table 8).

**Table 7:** Triclosan removal in wastewater treatment plants (Ying and Kookana 2007)

Level of treatment	Removal rate (%)
Primary treatment	2–96
Secondary treatment	
1. Trickling filter	58–96
2. Activated sludge	55–99
3. Activated sludge (simple treat)	61–72
Tertiary treatment	87–99

**Table 8:** Triclosan removal efficiencies (%) in selected drinking water treatment processes [adapted from NWRI (2010), Snyder et al. (2007)]

UV^a^	Chlorination^b^	Chloramination^c^	Ozonation^d^
50–80	80	80	95

^a^UV dose = 40 mJ/cm^2^

^b^Chlorine dose = 3 mg/l, contact time = 24 h

^c^Chloramine dose = 3 mg/l, contact time = 24 h

^d^Ozone dose = 2.5 mg/l, contact time = 2 min

In wastewater treatment plants employing membrane bioreactor, an estimated amount of over 90 % mass of triclosan is expected to have been removed from the water (Wijekoon et al. 2013). The high proportion of TCS reported to have been removed in wastewater treatment plants especially those plants which employ the conventional activated sludge process may be attributed to biodegradation under aerobic conditions (Bester 2003, 2005; Heidler and Halden 2007; Ying et al. 2007). Sludge treatment plants with biological treatment process showed the highest removal of TCS (Tohidi and Cai 2016). The abundance of bacterial TCS degraders namely, ammonia-oxidizing bacteria (AOB) and *Sphingopyxis* strain KCY1 in activated sludge systems has been reported (Lee and Chu 2015). It is thought that ammonia monooxygenase expressed by AOB is responsible for TCS degradation (Roh et al. 2009) while dioxygenase in the strain KCY1 co-metabolize TCS (Lee et al. 2012b). Sphingopyxis strain KCY1, a wastewater bacterium dechlorinates TCS presumably via 2,3-dioxygenase pathway (Lee et al. 2012b) producing androgenic metabolites (Lee et al. 2012a). *Trametes versicolor* and *Pycnoporus cinnabarinus*, species of white rot fungi which grow naturally on dead wood can degrade TCS. *Trametes versicolor* converts TCS into 2-*O*-(2,4,4′-trichlorodiphenyl ether)-β-D-xylopyranoside, 2-*O*-(2,4,4′-trichlorodiphenyl ether)-β-D-glycopyranoside, and 2,4-dichlorophenol whereas *Pycnoporus cinnabarinus* converts TCS into 2,4,4′-trichloro-2′-methoxydiphenyl ether and 2-*O*-(2,4,4′trichlorodiphenyl ether)-β-D-glycopyranoside (Hundt et al. 2000). The metabolites have been found less toxic but of lower microbiocidal activity than TCS (NICNAS 2009). *Pseudomonas putida* triRY and *Alcaligenes xylosoxidans* subsp. *denitrificans* TR1 have been reported to use TCS as their sole carbon source and clear particulate TCS from agar (Hundt et al. 2000), this can be made use of in bioremediation. Recent laboratory evidence showed that maximum degradation to 2,4-dichlorophenol and Cl^−^ by *Aspergillus versicolor* occurred at pH 5 and 7, depending on the substratum (Taştana and Dönmeza 2015). Minor (7 %) transformation products of TCS biodegradation namely, monohydroxy-, dihydroxy-triclosan derivatives and triclosan-*O*-sulfate in activated sludge have been reported recently (Chen et al. 2015). The hydroxylated derivatives have been reported earlier as products of triclosan transformation by *Sphingomonas* sp. PH-07 (Kim et al. 2011). The eco-toxicological significance of these minor transformation products appears obscure at this time. However, controversy still surrounds the impact of TCS on biodegradation of other co-pollutants in a given medium (Stasinakis et al. 2007; Svenningsen et al. 2011).

Chemical oxidants such as free chlorine (Canosa et al. 2005a), ozone (Chen et al. 2012b; Suarez et al. 2007), permanganate (Zhang and Huang 2003) and monochloramine (Wu et al. 2012b) are capable of degrading TCS in aquatic environment. Of all these oxidants, permanganate was reported as capable of degrading all the TCS in water under natural conditions (pH 8.1) and unlike other oxidants is not affected by the matrix (Wu et al. 2012b). TCS that persists in the effluents after activated sludge treatment may be chemically transformed after the discharge. Water disinfecting oxidant, sodium hypochlorite, a source of free chlorine is generally used and could chlorinate TCS, generating chlorinated derivatives such as 2,4-dichlorophenol and 2,4,6-trichlorophenol (Canosa et al. 2005a; Fiss et al. 2007). Ozonolysis appears to be the most efficient method of removing TCS in aqueous medium (Table 8). It generates, in addition to the chlorinated derivatives chloro-catechol, mono-hydroxy-triclosan and di-hydroxy-triclosan (Chen et al. 2012b) which are known to be more harmful than TCS to aquatic animals.

TCS is degradable under UV irradiation (photolysis) (Durán-Álvarez, et al. 2015; Lindstrom et al. 2002; Tixier et al. 2002). The UV-susceptibility in aqueous medium is related to the TCS large molar absorption coefficients (Carlson et al. 2015). Photolysis may be a significant route of TCS transformation in surface waters during summer. Laboratory evidence has shown that TCS is not appreciably photo-degraded in soils when compared to water samples (Durán-Álvarez, et al. 2015). It is believed that direct photolysis of TCS is hampered by the presence of organic substances which reflect incident photons (Hoigné et al. 1989). TCS photolysis may be enhanced in aqueous medium, by high (alkaline) pH in the presence of a sensitizer such as Fe(III) ions (Martínez-Zapata et al. 2013) or in the presence of surfactant in the aqueous medium which plays an accelerating role (Qiao et al. 2014). The photolysis produces 2,4-dichlorophenol and 2,4,6-trichlorophenol as stable TCS degradation products (Canosa et al. 2005b; Chen et al. 2012b; Fiss et al. 2007; Sanchez-Prado et al. 2006) while small amounts of derivatives of dioxin (2,8‐dichlorodibenzodioxin (DCDD) and furan (dichlorohydroxydibenzofuran) in the aqueous medium are produced as minor products (Latch et al. 2003, 2005; Sanchez-Prado et al. 2006; Son et al. 2009). These photoproducts are more toxic than the parent (TCS) compound (Sanchez-Prado et al. 2006).

About 5 % of TCS was reportedly transformed into methyltriclosan (MCS) by microbial activity (Bester 2003, 2005; Heidler and Halden 2007; Lozano et al. 2013). The final destination of TCS and its hydrophobic metabolites such as MCS is the sewage sludge (activated sludge and biosolids) through sorption wherein they are found in larger quantities than in the effluent aquatic medium (Chen et al. 2011; Heidler and Halden 2007; Kinney et al. 2008; McAvoy et al. 2002; Ying and Kookana 2007). Studies carried out on WWTP which included a Swiss plant showed that higher fraction of the TCS was adsorbed by the sludge than was present in the aqueous phase (Bester 2003; Singer et al. 2002). The work of Heidler and Halden (2007) seems to have confirmed this which reported an average of 80 % of TCS bound to the particulate matters. Later reports from mass balance studies (Lozano et al. 2013; Tohidi and Cai 2016) confirmed sorption of TCS on to the particulate matters and hence its removal from the aqueous phase. The report of studies by Lozano et al. 2013 on WWTP showed that most of the TCS is removed from aqueous phase during secondary treatment and nitrification–denitrification processes and no removal takes place during primary treatment process. We suggest systematic approach in the interpretation of results from mass balance studies on TCS removal in WWTP effluents in view of the reported over-estimation when comparing with field studies (Lozano et al. 2013).

Sludge is the final destination of both TCS and its primary metabolite, MCS. Reported TCS concentrations in sludges from different plants in Germany were from 1.4 µmol to 30.4 µmol/kg (Bester 2003) while the range in the United States was 1.8–53.9 µmol/kg (McAvoy et al. 2002) and 69.1 µmol–190 µmol/kg in biosolids reported elsewhere in the USA (Heidler and Halden 2007) (Table 9). These values are much higher when compared with the results presented by Azzouz and Ballesteros (2015) (Table 10) and may be related to increased usage of TCS. Both the parent compound and its metabolite find their way to the soil when it is amended with biosolids and are retained depending on the inter-play of biotic and abiotic factors (Butler et al. 2012). Clay has the least sorption for TCS while loamy soil the highest (Wu et al. 2009) and was reportedly more pronounced at top 10 cm soil layer and markedly occurs in summer (Butler et al. 2012). TCS sorption to soil may reduce in alkaline environment (Wu et al. 2009) releasing TCS to the aqueous medium.

**Table 9:** Triclosan concentrations in wastewater sediments and sludge (Dann and Hontela 2011)

Medium	Location	Concentration (nmol/kg)	References
*Sediment*			
Freshwater	Switzerland	180	Singer et al. (2002)
	Spain	nd–120	Morales et al. (2005)
Estuarine	USA	nd–2800	Miller et al. (2008)
Marine	Spain	9.3–450	Agüera et al. (2003)
*Sewage sludge*			
Activated sludge	USA	1.7–53.9	McAvoy et al. (2002)
	Spain	1.4–18.7	Morales et al. (2005)
	Germany	4.1	Bester (2003)
	Canada	2.1–5.0	Chu and Metcalfe (2007)
Biosolids	Australia	311–58,000	Ying and Kookana (2007)
	USA	36,300–103,600	Heidler and Halden (2007), Kinney et al. (2008), McClellan and Halden (2010)
	Spain	5210	Morales et al. (2005)
	Canada	2350–43,200	Lee and Peart (2002), Chu and Metcalfe (2007)

*nd* not detected

**Table 10:** Triclosan concentrations in agricultural soils, sewage sludge, river and pond sediments (Azzouz and Ballesteros 2015)

Medium	Location	Concentration (nmol/kg)
Agricultural soils (4 points, n = 12)	Spain	0.028–0.1
Pond sediments (2 points, n = 6)	Spain	0.15–0.2
River sediments (3 points, n = 9)	Spain	0–0.18
Sewage sludge (2 points, n = 6)	Spain	0.5–0.52

Soil samples from ten agricultural sites in Michigan previously amended with biosolids, collected over two years were shown to retain triclosan (0.55–3.5 nmol/kg) (Cha and Cupples 2009) while 0.31–24.4 µmol/kg triclosan was found in biosolids from three Michigan wastewater treatment plants. In certain dewatered municipal biosolids, 37.6 µmol/kg of TCS were reported (Gottschall et al. 2012) considered high when compared with previous values (Table 9). The practice of adding biosolids to agricultural soil has been reported in developed and developing countries (Table 10). In South Africa, six WWTP viz; Northern, Driefontein, Goudkoppie, Bushkoppie, Olifantsvlei and Ennerdale had been reported to generate a total of 91,611 tons of dry sludge per annum (Johannesburg Water 2015) which is used in composting by interested private farmers.

The concern is that biosolids may be an important source of TCS release to the environment as some 50 % mass of the incoming TCS in WWTP persist and are sequestered in the biosolids (Heidler and Halden 2007). The work of Waria et al. (2011) showed that TCS could be persistent in biosolids longer in fine sand (half-life 421 days) than silty clay loam (half-life 78 days) with MCS as a primary degradation product. TCS has been detected in soils amended with biosolids 33 years after application (Xia et al. 2010). A report (Gottschall et al. 2012) detected TCS in the soil about a year after application. This raises fear that the use of biosolids in farming even after treatment (Angin and Yaganoglu 2009) may recycle TCS and/its toxic metabolites, given its potential to persist (high estimated partition coefficient K_oc_ = 9200 and stability to hydrolysis) in soil, thereby exposing soil dwelling animals such as earthworms to TCS toxicity and at the same time surreptitiously increasing the human body burden of TCS via trophic levels. In anaerobic digester, exposure to oft-high level TCS concentrations has been recorded as leading to the proliferation of TCS-resistant genes (McNamara et al. 2014). Application of biosolids from such digester to soil could also lead to the release of resistant bacteria to the environment (Burch et al. 2014; Fahrenfeld et al. 2014).

Some authorities (NICNAS 2009; Ying and Kookana 2007) have attempted at determining the risk quotients associated with using effluents from sludge treatment plants in irrigation. However the kinetics of TCS in sewage sludges especially biosolids have not been intensively studied which continue to leave a gap in the knowledge about the amount of TCS present in the sludge and for how long. Further intensive research is also required beamed at elucidating the kinetics of degradation products of TCS such as the dioxin derivatives known for their toxicity and persistence.

## Triclosan bioaccumulation and ecotoxicity

Although TCS has the potential (log K_ow_ = 4.76) to bioaccumulate in fatty tissues, there is no evidence in human tissues. There is evidence that TCS and its metabolites bioaccumulate in mice (Kanetoshi et al. 1992) as well as in aquatic flora and fauna including algae, inverts and fish (Adolfsson-Erici et al. 2002; Buser et al. 2006; Capdevielle et al. 2008; Coogan and La Point 2008). TCS concentrations as high as 276.3 µmol/kg has been reported in the bile of fish (*Abramis brama*) (Houtman et al. 2004). Consequently toxic endpoints attributable to TCS have been recorded in these organisms. IC_25_ of 0.55 µmol/kg affected hatchability in Zebrafish *Danio rerio* after 9 days exposure (Tatarazako et al. 2004). The swimming ability of fathead minnow (*Pimephales promelas*) was reduced when exposed to ≥0.52 μM TCS for 7 days (Cherednichenko et al. 2012) but the exposure level used in the study might be questionable as being environmentally irrelevant because TCS levels in surface waters normally occur at nanomolar levels (Table 3).

Microalgae and *Hydra magnipapillata* are important members of prisere in aquatic ecosystem. Microalgae communities are particularly sensitive to TCS at effective concentrations of about 0.035 nM (Wilson et al. 2009). It has been reported that at concentration (EC_10_) of 3.4 nM for 96 h exposure, TCS affected biomass of *Anabaena flos*-*aquae* the blue-green alga (Orvos et al. 2002). Among benthic microbial communities, TCS was found to act as a selective factor favouring the growth of cyanobacteria over algae (Drury et al. 2013). The ecotoxicological implication is that cyanobacteria produce toxins which affect zooplanktons and which in turn threatens the survival of higher members of the trophic levels (Bláha et al. 2009). Exposure of *Hydra magnipapillata* to TCS (3.5 µM for 4 h), resulted in epidermal tissue and nematocyst damage (Park and Yeo 2012). IC_25_ of 0.55 µmol/kg affected hatchability in Zebrafish *Danio rerio* after 9 days exposure (Tatarazako et al. 2004). Plants and bacteria are thought to share the same fatty acids synthesis pathways; and experiments conducted with *Arabidopsis* family *Brassicaceae* have shown that enoyl-acyl carrier protein reductase is a common target of TCS (Serrano et al. 2007), probably implying that it is toxic to plants as much as to bacteria.

Methyltriclosan (MCS) is a metabolite of TCS formed by bacterial methylation (Bester 2003, 2005). Its presence in aquatic animals first pronounced by Miyazaki et al. (1984) is known to accumulate in aquatic animals than TCS presumably because of its higher partition coefficient (log K_ow_ = 5; log K_oc_ = 4.1); compare TCS (log K_ow_: 4.2–4.8; log K_oc_: 4.3) (Chen et al. 2011). MCS concentrations in lake fish have been reported to be between 4 and 370 ng/g (Balmer et al. 2004). Higher concentrations of 520–596 μg/kg wet weight were reported in fresh water fish such as *Cyprinus carpio* (Leiker et al. 2009).

The negative impact of TCS on ecosystem is expected to have economic consequences. The United Nations Food and Agriculture Organization (FAO) reports (FAO 2014) that about 25 million tonnes of seaweeds and other algae have been harvested annually for use as food, in cosmetics and for fertilizers, and are processed into thickening agents or animal feed additives. Given the negative effects of TCS on the aquatic flora and fauna such as algae and fish, all the economic advantages such as protein supply from water resources as well as employment provision, risk shortages if TCS circulation is not properly regulated.

## Triclosan antimicrobial activity

The antimicrobial activity of TCS spans against Gram positive and Gram negative non-sporulating bacteria, some fungi (Schweizer 2001), *Plasmodium falciparum* and *Toxoplasma gondii* (Al-Doori et al. 2003). At low concentrations TCS is bacteriostatic which is predicated on its inhibitory effect on the bacterial enoyl acyl carrier protein reductase (Heath et al. 1999) of fatty acid elongation pathway, whereas at higher concentrations (as found in dermatological preparations) it has bactericidal effect through membrane intercalation and triclosan-induced K^+^ leakage (Escalada et al. 2005; Russell 2004; Villalain et al. 2001). Staphylococci, some Streptococci, some Mycobacteria, *Escherichia coli* and *Proteus* spp, Methicillin-resistant *Staphylococcus aureus* (MRSA) strains are all sensitive to TCS (Al-Doori et al. 2003; Suller and Russell 1999). TCS minimum inhibitory concentration of 500 ppb as an effective bactericide in the products has been reported. Showering or bathing with 2 % (w/w) TCS has been shown to be an effective concentration for the decolonization of patients whose skin is carrying MRSA (Tuffnell et al. 1987). TCS-based products have been used successfully to control MRSA (Brady et al. 1990; Zafar et al. 1995). Enterococci are much less susceptible than Staphylococci to TCS while *Pseudomonas aeruginosa* is highly resistant (Russell 2003) to it.

Microbial resistance to TCS has been reported (McMurry et al. 1998; Sanchez et al. 2005) in which membrane impermeability and efflux pump are thought to play a major mechanistic role (Gomez-Escalda et al. 2001; Sanchez et al. 2005). Studies conducted under environmentally relevant TCS concentrations produced TCS resistance (Nietch and Quinlan 2013). In the same vein, urbanization with regular discharge of TCS into the surface waters has been reported to favour TCS resistance among benthic microbial communities (Drury et al. 2013). The report of McNamara and co-workers (2014) on anaerobic digesters showed that the structure of the microbial community as well as TCS concentration is critical to the selection of resistance strains. Recent report (Gantzhorn et al. 2015) has demonstrated that mutation of one of the sigma factors (transcription initiation factor required for RNA polymerase recognition of its promoter) in combination with that of *fab I* (encodes the enoyl-acyl carrier protein reductase), confers high-level microbial resistance to TCS. It however remains to be elucidated the precise role of the sigma factors, in particular which of the factors, for example *rpoS* or *rpoD* is responsible for the mutation. Microbial resistance to TCS may lead to the worst scenario of the appearance of the antibiotic insensitive bacteria the so called super-bugs, as well as cross-resistance to other antibiotics such as isoniazid and ethionamide (an anti-tuberculosis pro-drugs), which target *Mycobacterium tuberculosis* enoyl acyl carrier protein reductase (InhA) (Freundlich et al. 2009) underscoring the need to regulate TCS with a view to preventing over-use. The precise role of TCS on the selection of antibiotic resistance genes as well as multidrug resistance genes in the environment needs be determined. The concentration of TCS that is required for resistance selection in environmental communities needs be worked out too.

## Conclusion and future directions

Available data on the occurrence of TCS in various environmental media, in human body and in wildlife show that the compound is not well regulated. Its uncoordinated use and careless disposal may threaten lives and the ecosystem generally. Cell based studies have shown toxicity potentials of TCS in a number of cells. Results of epidemiological studies as enunciated in this review may have supported the in vitro study reports. Cell-based assays are short-term (hours–days) and cannot be used to directly address the effect of chronic exposures. Pursuant to this widely held criticism, additional data need be generated from in vitro and in vivo studies as well as carefully designed epidemiological studies in order to make a conclusive remark on the role of the chemical in health and disease.

The detection of TCS in human fluids and tissues may not be an indicator of long term exposure as available data are insufficient to confirm its bio-accumulation in tissues (Geens et al. 2012), more so it is thought that TCS inhibits enzymes responsible for its metabolism (conjugation) (Wang et al. 2004). There is presently paucity of data on both pharmacodynamics and pharmacokinetics of TCS. Sufficient data would provide leeway in understanding the toxicity of TCS. The toxicological significance of the inhibitory effect of TCS on human fatty acid synthase (Liu et al. 2002) is not well understood though its anti-proliferative effect has been reported in some cancer cells (Honkisz et al. 2012), however administration of TCS orally to rats, dogs and baboons was reported to produce minimum toxicity in these animals (Bhargava and Leonard 1996). The presence of detectable levels of TCS in human tissues fuels the suspicion that the compound may impact negatively on human physiology. Its adverse effect on innate immunity has been reported and so are clinical reports purporting its management of human allergic skin disease (Sporik and Kemp 1997; Tan et al. 2010). Certain epidemiological studies have attempted to answer this question but sufficient consideration was not usually given to such confounding factors as inter-individual variability.

The precise role of TCS on the selection of antibiotic resistance genes as well as multidrug resistance genes in the environment needs be elucidated. The concentration of TCS that is required for resistance selection in environmental communities also needs be worked out. The relationship between TCS exposure and bio-accumulation in terrestrial animals is still inconclusive (Higgins et al. 2011) and would require expansive work which should include the kinetics of TCS conversion in the soils to its primary metabolite and uptake by such terrestrial animals as earthworms and snails which among others are important in agriculture and nutrition.
